# The theragnostic advances of exosomes in managing leukaemia

**DOI:** 10.1111/jcmm.70052

**Published:** 2024-12-10

**Authors:** Subhrojyoti Ghosh, Anuvab Dey, Aneshwa Chakrabarti, Tiyasa Bhuniya, Neelparna Indu, Anirban Hait, Ankita Chowdhury, Aritra Paul, Atharva A. Mahajan, Marios Papadakis, Athanasios Alexiou, Saurabh Kumar Jha

**Affiliations:** ^1^ Department of Biotechnology Indian Institute of Technology Madras Chennai Tamil Nadu India; ^2^ Department of Biosciences and Bioengineering Indian Institute of Technology Guwahati Guwahati Assam India; ^3^ Department of Chemistry and Chemical Biology Indian Institute of Technology, Indian School of Mines Dhanbad Dhanbad India; ^4^ Department of Biotechnology NIT Durgapur Durgapur West Bengal India; ^5^ Department of Biotechnology Heritage Institute of Technology Kolkata India; ^6^ GenoPhe Biotech Pvt. Ltd. Bengaluru Karnataka India; ^7^ Department of Surgery II University Hospital Witten‐Herdecke Wuppertal Germany; ^8^ University Centre for Research & Development Chandigarh University Mohali Punjab India; ^9^ Department of Research & Development Funogen Athens Greece; ^10^ Department of Research & Development AFNP Med Wien Austria; ^11^ Department of Science and Engineering Novel Global Community Educational Foundation Hebersham New South Wales Australia; ^12^ Department of Zoology, Kalindi College University of Delhi Delhi India

**Keywords:** biogenesis, drug resistance, exosomes, leukaemia, therapeutics

## Abstract

Leukaemia, a group of haematological malignancies, presents ongoing diagnosis, prognosis, and treatment challenges. A major obstacle in treating this disease is the development of drug resistance. Overcoming drug resistance poses a significant barrier to effective leukaemia treatment. The emergence of exosome research has unveiled new insights into the probable theragnostic implementations in leukaemia. Various research has exhibited the diagnostic possibilities of exosomes in identifying leukaemia‐specific biomarkers, including genetic mutations and fusion transcripts. Additionally, exosomes have been implicated in disease progression and treatment response, rendering them appealing targets for therapeutics. Exosomes, originating from diverse cell types, are instrumental in intercellular communication as they participate in the functional transportation of molecules like proteins, nucleic acids and lipids across space. Exosomes have a dual role in immune regulation, mediating immune suppression and modulating anti‐leukaemia immune responses. Interestingly, exosomes can even act as drug transport vehicles. This review delves into the intricate process of exosome biogenesis, shedding light on their formation and release from donor cells. The key mechanisms engaged in exosome biogenesis, for instance, the endosomal sorting complexes required for transport (ESCRT) machinery and ESCRT‐independent pathways, are thoroughly discussed. Looking ahead, future approaches that leverage innovative technologies hold the promise of revolutionizing disease management and improving patient outcomes.

## 1 INTRODUCTION

Exosome, a variety of extracellular vesicles (EV), was first identified in 1983. The term “EV”, as defined by the Minimum Information for Studies of EV (MISEV) guidelines, refers to a broad category of cellular structures that are derived from biological cells through a natural process. These structures lack a nucleus and are found outside the cells from which they originated. EVs encompass various types of vesicles, such as exosomes and microvesicles, which are involved in intercellular communication and can carry diverse biomolecules.^1^ When researchers define a specific subtype of EVs, they primarily consider the physical characteristics of the cells involved. A notable study by Harding, Heuser and Stahl unexpectedly discovered a type of late endosome called a “multivesicular endosome (MVE)”. These MVEs fuse with the plasma membrane, releasing tiny vesicles within them.^2^ Subsequent research by Pan and Johnstone demonstrated that the transferrin receptor is transported outside the cell through these vesicles in sheep reticulocytes. Five years later, Rose Johnstone coined the term “exosome” to refer to these vesicles. The process of exocytosis, tightly regulated by the cell, releases exosomes composed of two lipid layers ranging in diameter from 30 to 150 nm.^3^ Previously, it was assumed that exosomes discard cellular waste. Still, recent research has produced convincing evidence that they contain various biomolecules that can govern cellular metabolism and manifest as changed physiological or pathological processes. These exosome‐effector chemicals may serve as potential biomarkers for particular disorders.^4^ The meaning of “exosome” has thus changed since it was discovered. Exosomes are diverse and can even mimic original cells.^5^ It mainly comprises the molecular components of the original cell. It has been established that exosomes are complex in almost all diseases.^6^ For example, the secretion of exosomes is useful in eradicating harmful cytoplasmic DNA from cells to maintain cellular homeostasis.^7^ Concurrently, owing to the high heterogeneity of inclusion bodies, exosomes behave like a sword with two edges in disease initiation, proliferation and suppression.^8^ Thus, exosomes have been rightfully considered diagnostic biomarkers,^9^ delivery platforms, therapeutic targets,^10^ and therapeutic agents for various diseases. Exosomes, a disease‐targeted delivery platform, have been rapidly developed with impressive results^11^ and are regarded as the next‐generation advanced nanotechnology theragnostic platform. Exosomes' potential roles have evolved over time from being waste transporters to being used for intracellular communication and medicinal applications. Exosome research has experienced significant expansion, and it is currently regarded as a crucial cellular communication method that delivers its content to the destination cell. Additionally, advancements in exosome research have shown that they regulate multiple biological processes, including cellular differentiation, apoptosis, the release of biological mediators and maintenance of homeostasis, along with showing other therapeutic and diagnostic effects.^12^ Exosomes make a special contribution to the biology of cancer. Evidence suggests that exosomes produced by most cancer cells are responsible for metastasis, treatment resistance, tumour immune emergence and tumour growth. This makes them recognized as a cancer biomarker after studies revealed increased expression of exosomal miRNAs in the blood plasma of most individuals suffering from cancer.

## 2 BIOGENESIS

**FIGURE 1:**
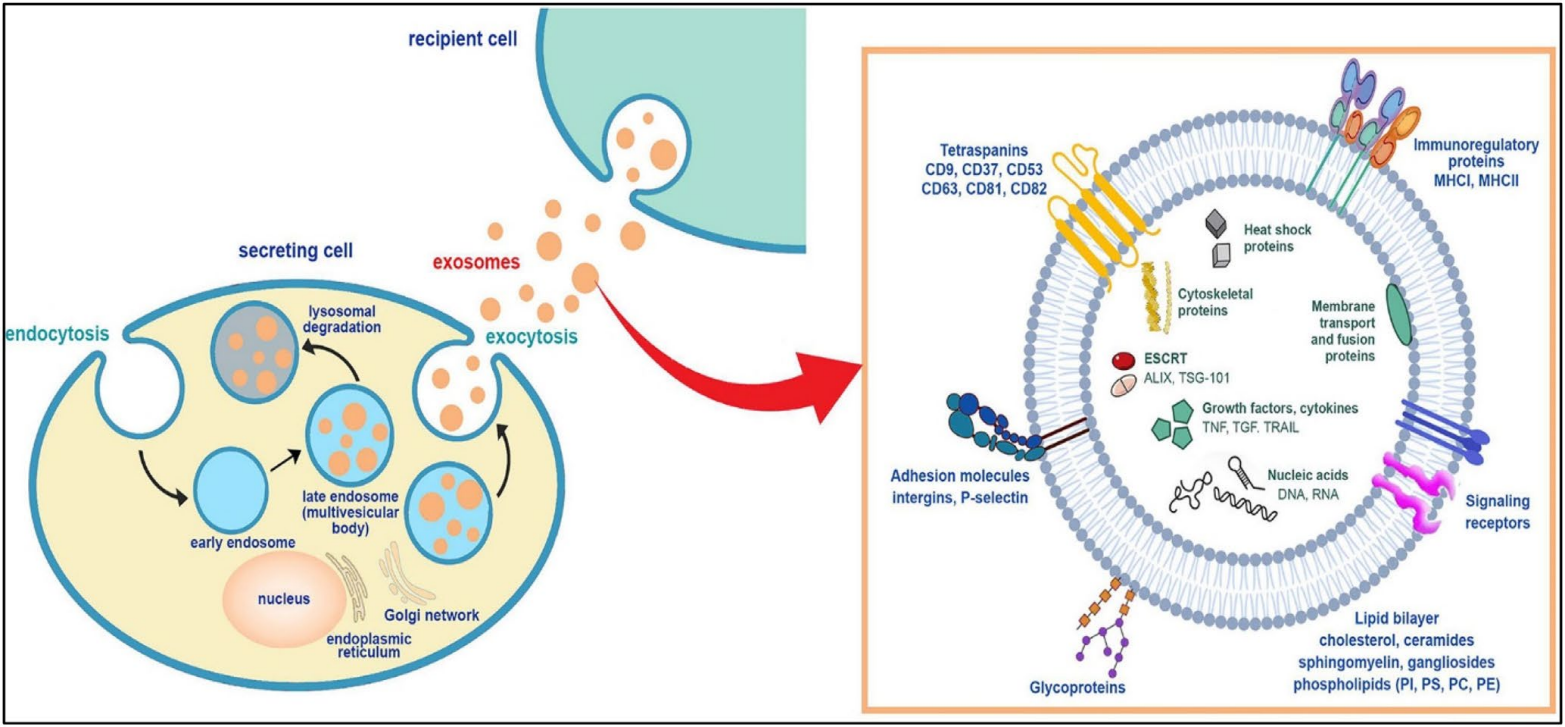
Exosome Biogenesis (Adapted with permission from ref^23^ Copyright© 2022 American Chemical Society).

EVs are membrane vesicles of nanoscale that cells actively discharge. MVEs can take shape through two distinct pathways: the exosomal pathway, where the inner membrane buds inward, and the microvesicle pathway, where the plasma membrane buds outwards.^13^ Multivesicular bodies (MVBs) are the vesicles created when the plasma membrane sprouts outward. Exosomes are dynamic substances continuously produced by the cell's endosomal system and exposed to the external environment through exocytosis.^14^ The late endosomal system is formed when the multivesicular body membrane invaginates, extending the late endosomes in the crease to create intraluminal vesicles (ILVs).^15^ Several particular proteins are incorporated into the vesicles during the production of the ILVs. These vesicles, or exosomes, fuse with the cell's plasma or outer membrane.^16^ Exosomes are formed in the endocytic pathway. The initial stage of endocytosis involves the infolding of the cell membrane that produces an early endosome. The outer membrane of the late endosome then grows externally to generate small vesicles that play a crucial role in forming MVBs.^17^ The conformation of ILVs distinguishes the MVB following the inward creasing of the inner body membrane. Lysosomes cause the breakdown of these constituents, whereas exosomes are a widely used term for various ILVs discharged into the extracellular environment.^18^ When observed under transmission electron microscopy, typical exosomes appear as spherical structures, but they exhibit a distinctive biconcave or cup‐shaped appearance when generated through suspension‐based methods. Numerous research works have demonstrated the significance of lipids in exosome genesis. In a recent inquiry, it was postulated that phospholipase activity could potentially regulate exosome biogenesis and ILV formation.^19^ Diacylglycerol kinase modulates the deployment of exosomes from T cells.^20^ Distinct exosomes have distinct roles for different types of proteins. The ESCRT is involved in leading cargo into ILV. ESCRRT includes PDCD6IP (ALIX), TSG101, HRS, CD9, CD82, etc. Other proteins are PLG2, DGKα, etc.^21^ Subsequent to the merging of multivesicular bodies (MVBs) with the cell membrane; exosomes are deployed via a process that involves small GTPases like RAB27B, RAB7, RAB11, RAB27A, RAB31 and RAB35 in certain cells. SNARES family proteins such as VAMP7 and YKT6 are also involved in this mechanism. Although some steps may be similar, variations in this process, including cell type, can ascertain the exosome secretion of different subpopulations.^22^ (Refer to Figure 1).

## 3 EXOSOME IN IMMUNE SUPPRESSION

**FIGURE 2:**
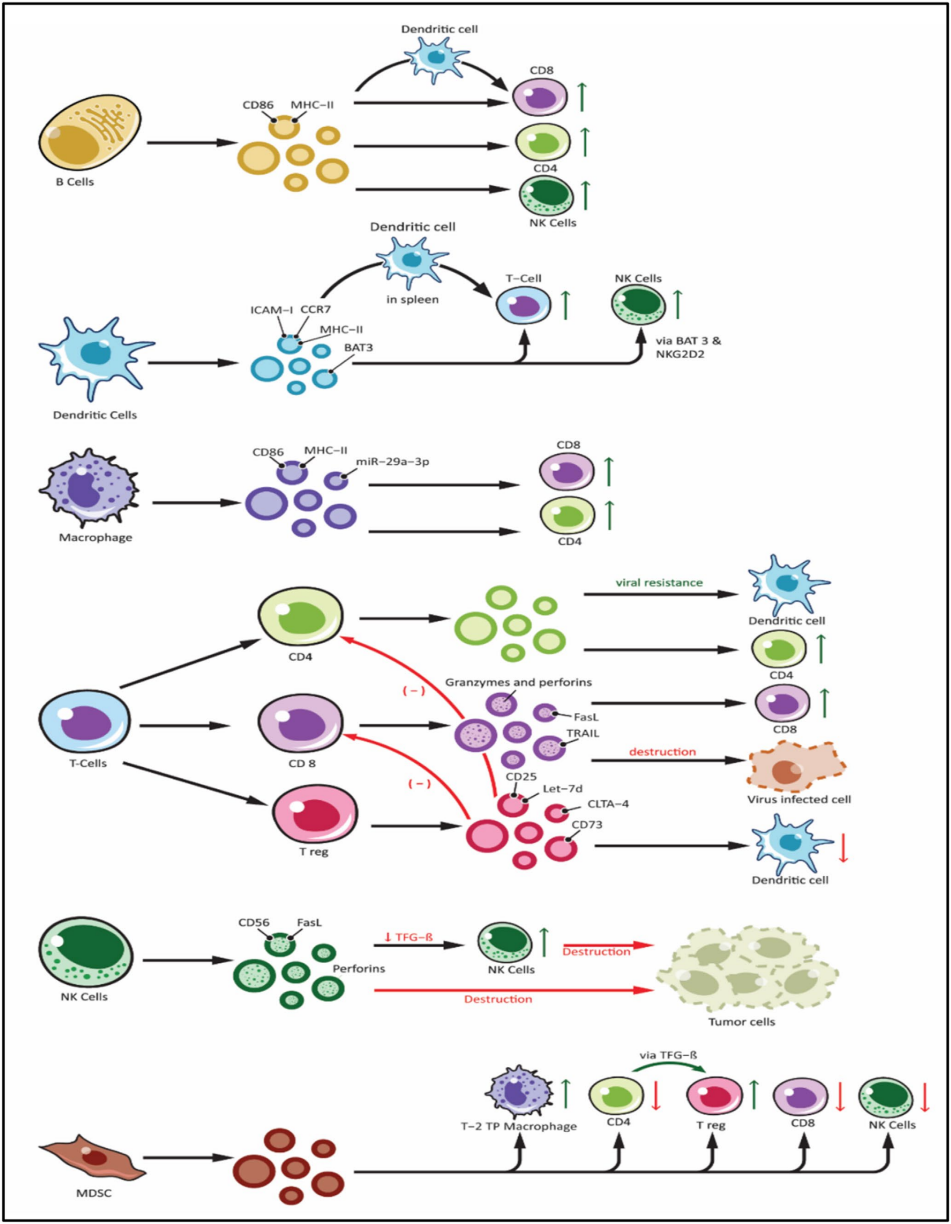
The impact of exosomes derived from immune cells on the cellular components of the immune system is noteworthy. These exosomes originate from various immune cells such as B‐lymphocytes (B cells), T‐lymphocytes (including CD4+, CD8+ and Treg subsets), Natural Killer cells (NK‐cells), Dendritic cells, other antigen‐presenting cells (APCs) and Myeloid‐Derived Suppressor Cells (MDSC). Immune cell‐derived exosomes can influence various other cells involved in immune responses. Exosomes can stimulate the immune response by presenting peptide–MHC complexes to T cells, transferring antigens to other dendritic cells, and activating T cells, B cells and NK cells. Likewise, exosomes from Treg and NK cells exhibit inhibitory functions, which include suppressing the adaptive arm of the immune response and inducing the destruction of tumour cells by NK cells. Exosomes derived from MDSC also play a significant role in immune suppression, primarily by activating Treg and promoting a Type‐2 Tumour promoting phenotype in macrophages (T‐2 TP macrophages) (Adapted with permission from ref.^59^ Copyright © 2022, The Author(s)).

Cancer immunosurveillance is a natural process by which the immune system controls cancer growth in its early stages.^24^, ^25^ However, tumour cells use clever escape mechanisms as cancer progresses to evade immune detection and activate immunosuppressive pathways, resulting in failed immune surveillance.^26^ Cancer cells can alter the microenvironment and act on the functioning of the immune system through various mechanisms. These include direct cell‐to‐cell contact along with the release of soluble factors that can suppress the immune response by altering myeloid differentiation.^27^ An emerging and novel mechanism of immune suppression involves the active release of immune‐suppressive microvesicles called TDEs by tumour cells.^28^ EVs released by tumours, known as TDEs, can act as influential promoters of tumour immunosuppression (Refer to Figure 2). They do this by serving as a transport mechanism that is relatively resistant to degradation, allowing for more efficient propagation of signals that promote immune tolerance from the tumour site to other parts of the body.

The cargo of TDEs includes components that cause malfunction of immune cells in different manners, inhibiting the immune response against tumours.^29^ In the beginning, TDEs attach to the immune cells via ligands or antigens identified by particular lymphocyte receptors. Subsequently, TDEs rapidly combine with the cell surface membrane, facilitating the discharge of their contents into the cytoplasm. TDEs can be swiftly absorbed and internalized by phagocytic cells, like macrophages and DCs. On the other hand, T cells seem to have a lower capacity to take up TDEs. Instead, TDEs converse with the molecules present on the surface to transmit signals that trigger long‐lasting calcium influx and downstream signalling molecule activation. This, in turn, alters the genetic makeup of the receiving cell.^30^

### 3.1 T cells

TDE may transfer the ligands present on their surface to T cell surface receptors without being internalized to influence T cell activities and gene expression.^31^ One of the mechanisms for the immunological escape of malignancies is the attachment of planned cell death ligand 1 (PD‐L1) to its receptor, programmed cell death protein 1 (PD‐1), which can result in the deactivation of cytotoxic T cells.^32^ In addition to the surface ligand interaction mentioned above, the internalization strategy used by the TDE protein cargo might disrupt T cell activities. It has been claimed that tumour antigens that express TDE can prevent T‐cell activation and cause T cells to die. According to studies in mice, exosome transfer from tumour‐bearing mice to animals inoculated with ovalbumin reduced the action and number of antigen‐specific T lymphocytes.^33^ According to Whiteside et al., TDE boosted CD4^+^ T cells ex vivo while inhibiting the growth of human CD8^+^ T cells.^29^ Moreover, TDE causes immune suppression by promoting the death of antitumor CD8^+^ effector T cells and boosting the suppressor action of CD4^+^ T regulatory cells, aiding in tumour escape.^34^ According to Miyazaki et al., TDE produced by prostate cancer cells that overexpress EBAG9 can help malignancies escape the immune system by preventing T‐cell cytotoxicity and altering the expression of immune‐related genes in T cells.^35^

### 3.2 Natural killer (NK) cells

NK cells are inborn lymphoid cells that control homeostasis by destroying activated immune cells and defending the host against disease and malignant cells.^36^ Compared to healthy people, the number and activity of NK cells are lower in individuals suffering from cancer. According to reports, TDE inhibits the activity of NK cells, which helps cancer cells escape the immune system. Recent research has shown that TDEs can significantly impact tumour development and progression. A journal recently published a research article demonstrating that TDEs generated by 4 T.1 murine mammary tumour cells stimulated the proliferation of transplanted tumour cells in both syngeneic BALB/c mice and nude mice. These findings suggest that TDEs could be instrumental in modulating the immune response to tumours and might be a promising target for therapeutic intervention. Further investigation into the mechanisms behind TDE‐mediated tumour promotion could lead to novel approaches in cancer treatment. Our findings have led us to understand the complex interaction between tumours and the body's defence mechanism in a better fashion. These might profoundly impact the future of cancer research and therapy.^37^ Mechanistically, pretreatment with TDE may assist tumour growth by preventing NK cell activation by IL‐2 and the cytotoxic response of these cells to tumour cells.^37^, ^38^ According to Lundholm et al., research on prostate tumour‐derived TDE that expresses the NKG2D ligand preferentially downregulates NKG2D on NK and CD8+ T cells, impairing cytotoxic activity in vitro.^39^ With TDE cells made from the cell lines K562 and IGR‐Heu, Berchem et al. elucidated that oxygen deprivation causes a dramatic rise in TGF‐ levels. The intricate interplay between hypoxic TDEs and NK cells has revealed a fascinating mechanism: TDEs infused TGF‐1 into NK cells, downregulating the pivotal activating receptor NKG2D and a consequential impairment of NK cell activity. This discovery highlights the pivotal act of TDEs in orchestrating the tumour microenvironment and points towards new avenues for cancer immunotherapy.^40^ Following microRNA profiling, it was seen that TDEs obtained from cancer cells under hypoxic conditions contained significant levels of miR‐210 and miR‐23a.The powerful cytotoxic ability of NK cells was no match for the insidious effects of hypoxic TDEs, which infiltrated the NK cells and caused a significant reduction in the expression of CD107a, a well‐known marker of the functional activity of NK Cells. The once‐mighty immune system warriors were left hampered and powerless, unable to execute their vital function of seeking and destroying invading pathogens.^40^

### 3.3 Macrophages

Macrophages are powerful immune cells that play a variety of roles in the inflammatory process, including antigen presentation, phagocytosis and immunomodulation. Their functional phenotypes are extremely adaptable and vary depending on the signals in their microenvironment, making them key players in the immune response.^41^ In the tumour microenvironment, tumour‐associated macrophages (TAMs) can be influenced by intercellular communication with cancer cells via TDEs.^42^ Research has demonstrated that TDEs secreted by breast cancer cells can activate the NF‐B signalling pathway in macrophages. This effect is mainly achieved through the interaction of palmitoylated surface protein ligands of TDEs with TLR2 receptors present on the surface macrophages. Moreover, Annexin A2, which is hugely expressed in TDEs found in breast cancer, has been shown to prompt angiogenesis by activating p38MAPK, NF‐B and STAT3 pathways interceded by macrophages.^43^ These findings reciprocate the importance of TDE‐mediated communication in regulating the phenotype and action of macrophages in the TME, providing new insights into potential therapeutic targets for cancer treatment.

### 3.4 Dendritic cells (DCs)

DCs are the master orchestrators of the body's defence mechanism, serving as the gatekeepers of adaptive immunity. These antigen‐presenting cells (APCs) are essential in initiating the immune response by recognizing, processing and putting the antigens on their surface to T cells via co‐stimulatory molecules, MHC molecules and cytokines. However, cancer cells have found a way to hijack this process and evade the immune system through TDEs.^44^

Recent studies have shown that TDEs can effectively inhibit DC differentiation, causing a ripple effect on the immune system's ability to instigate a response against cancer. TDEs contain functional proteins and miRNAs that can modulate DC maturation and migration, causing them to undergo apoptosis and preventing them from presenting antigens to T cells. Inhibition of PD‐L1 has been shown to restore some of the immunosuppressive abilities of TDE‐treated DCs, suggesting a potential therapeutic target.^45^, ^46^

Although TDEs are known to induce immune dysfunction, they also carry donor antigens that have the potential to initiate specific cytotoxic T lymphocyte (CTL) activities in vitro or in vivo, suggesting that TDEs could be utilized as a vaccine against cancer. In a study by Andre et al., it was shown that TDE antigens could be internalized and cross‐presented by HLA‐A2+ monocyte‐derived DCs via MHC‐I molecules, proposing an optimistic route in the development of cancer immunotherapy.^47^

These findings illuminate the crucial role of TDEs in modulating DC function and shaping the immune response. Further research on the complex interplay between TDEs and DCs could unlock new opportunities for cancer immunotherapy, ultimately leading to better outcomes for cancer patients.^48^

### 3.5 Myeloid‐derived suppressor cells (MDSCs)

MDSCs are essential to immune suppression during pathological conditions, particularly cancer. MDSCs are myeloid cells (they are not matured) that comprise DCs, macrophages, and granulocyte precursors and their differentiation and maturation are blocked in cancer patients, leading to their accumulation in vivo.^48^, ^49^ The accumulation of MDSCs in cancer patients has been linked to impaired immune function, which hinders antigen processing, presentation and T‐cell activation, suppressing immune activity and immune responses that resist tumour formation. Recent studies have suggested that TDEs released by tumours are very important in the survival, development and immune suppression of MDSCs in the tumour microenvironment. The contents of TDEs are diverse, but their protein and miRNA components are vital in regulating the cellular processes of MDSCs.^50^, ^51^

Studies have shown that TDEs interact with TLR2/MyD88 on MDSCs via their membrane HSP ligands, leading to MDSC activation. Furthermore, TDE proteins have been extensively studied in relation to MDSC expansion and immuno‐suppression. Research works have shown that TDEs are internalized by myeloid cells, which are obtained from bone marrow (BM), leading to the accumulation of MDSCs that express various molecules, including VEGF, Cox2, IL‐6, and Arg1. These MDSCs contribute to the advancement of tumours by promoting the TGF and PGE2‐ molecules.^52^, ^53^, ^54^

Moreover, TDEs allow for the straight movement of nucleic acids, particularly RNAs, engaged in cell–cell communication. Studies have shown that MDSCs are the primary recipients of TDE‐nucleic acids. After internalizing labelled TDEs, MDSCs display over‐expression of suppressive molecules and anomalous miRNA‐expressing profiles, including aberrant expression of miR‐320, miR‐342‐3p, miR‐126‐3p, miR‐27b, all of which have been implied in tumour progression.^55^, ^56^ These findings highlight the crucial role of TDEs in promoting MDSC expansion and immuno‐suppression in the TME and suggest that targeting TDE‐mediated MDSC regulation could be a propitious strategy for cancer therapy.^57^, ^58^

## 4 LEUKAEMIA TYPES AND EXOSOMES

Leukaemia is a type of cancer that affects the blood and BM, characterized by the overproduction of abnormal white blood cells. These abnormal cells can crowd out healthy blood cells, leading to various health problems. Leukaemia is classified based on how quickly it progresses and the type of white blood cell it affects.^60^ In leukaemia, exosomes are released primarily by the malignant leukaemic cells, which can be either myeloid or lymphoid in origin, depending on the type of leukaemia. These exosomes are involved in various processes that promote disease progression, communication with the microenvironment and immune system evasion.^60^ The following sections give a comprehensive description of the types of leukaemia cells that release exosomes:

### 4.1 Acute myeloid leukaemia (AML) & exosomes

*Myeloid Blast Cells*: In AML, immature myeloid cells (myeloblasts) proliferate uncontrollably. These myeloid blast cells are a primary source of exosomes in AML. The exosomes from these cells can carry oncogenic factors, drug‐resistance proteins and other molecules contributing to the aggressive nature of AML.^61^

### 4.2 Acute lymphoblastic leukaemia (ALL) & exosomes

*Lymphoid Blast Cells*: In ALL, the cancer originates in immature lymphoid cells (lymphoblasts). These lymphoblasts release exosomes that can influence the BM microenvironment, promote survival and proliferation of leukaemia cells and modulate the immune response.^62^

### 4.3 Chronic myeloid leukaemia (CML) & exosomes

*Myeloid Progenitor Cells*: In CML, the disease is characterized by the proliferation of myeloid progenitor cells, which carry the Philadelphia chromosome. These cells release exosomes that can play a role in disease progression and resistance to therapy.^63^

### 4.4 Chronic lymphocytic leukaemia (CLL) & exosomes

*Mature B Lymphocytes*: In CLL, the malignant cells are typically mature B lymphocytes. These cells release exosomes that can carry immunosuppressive molecules, aiding the leukaemia cells in evading the immune system.^64^

### 4.5 Other leukaemia types & exosomes

*Hairy Cell Leukaemia and Others*: Less common types of leukaemia, such as hairy cell leukaemia, also involve malignant cells that release exosomes. These exosomes have similar roles in promoting disease progression and modulating the immune response.

## 5 ANGIOGENESIS

Angiogenesis means the production of new blood vessels, which is, in turn, modulated by chemical signals. During this process, endothelial cells (ECs), present on the interior surface of blood vessels, undergo proliferation, movement and differentiation. The development and metastasis of tumours heavily depend on angiogenesis.^65^ It has been established that TDEs have a transitional part to play during this process.^66^, ^67^ TDEs deliver pro‐angiogenic chemicals to ECs, improving angiogenesis through growth factors like VEGF, NO, FGF and Ang.^68^ Through blood circulation, exosomes produced by leukaemia cells spread to other organs, including lymph nodes (LN). Furthermore, clinical investigations have indicated that miRNAs are critically involved in the growth of tumours.^68^ A study on imatinib‐resistant and LAMA84 cells showed that exosomes directly affect ECs, modulating neovascularization. Moreover, LAMA84 exosomes also have miR‐126, which targets VEGF signalling regulators implicated in angiogenesis.^69^ Another study reveals that exosomes produced by CLL enhance stromal cell relocation, multiplication, and liberation of inflammatory cytokines and endothelial cell growth can accelerate tumour development.^70^ According to sources, CML‐derived exosomes carrying miR‐92a have been found to instigate human umbilical vein ECs (HUVECs) to take the shape of tubular structures. This is believed to occur through the activation of Src signalling. Umezu et al. reported the communication between leukaemia cells and ECs through exosomal miRNA, which might play an important role in developing new blood vessels in leukaemia. In short, pro‐angiogenic growth factors found in leukaemia's exosomes allow leukaemia's multiplication and metastasis to distant regions by promoting angiogenesis.^71^, ^72^

## 6 EXTRACELLULAR MATRIX (ECM)

The ECM is a mesh of interlinked macromolecules that acts as a scaffold for the cells found in tissues and organs.^73^ Cells are subject to changes in their characteristics due to the surrounding microenvironment, which ultimately influences their capacity to migrate, multiply and thrive.^74^, ^75^ ECM‐degrading enzymes, known as Matrikines, are deployed in response to physiological and pathological events to reconstruct the ECM, restore a suitable functional meshwork and preserve tissue homeostasis.^75^, ^76^ The hijacking of ECM remodelling during cancer metastasis results in stromal tumorigenesis.^77^, ^78^, ^79^ Cancer cells' invasive and metastatic activities involve various important ECM components, including proteoglycans, collagen, laminins, fibronectin, elastin, other glycoproteins and proteinases.^80^ Versican is a hyalectan that is found in the interstitial ECM. Its EGF‐like repeats trigger EGFR signalling, promoting cancer cell development and invasion. Another element of the ECM, chondroitin‐SO_4_ proteoglycan 4 (CSPG4), is crucial in maintaining the interconnections among the cells in the ECM matrix. An ECM protein called Lumican controls fibril organization and circumferential growth. The migration of epithelial cells and tissue healing are all significantly influenced by it.^80^

## 7 METASTASIS (EMT, ORGAN‐SPECIFIC METASTASIS)

**FIGURE 3:**
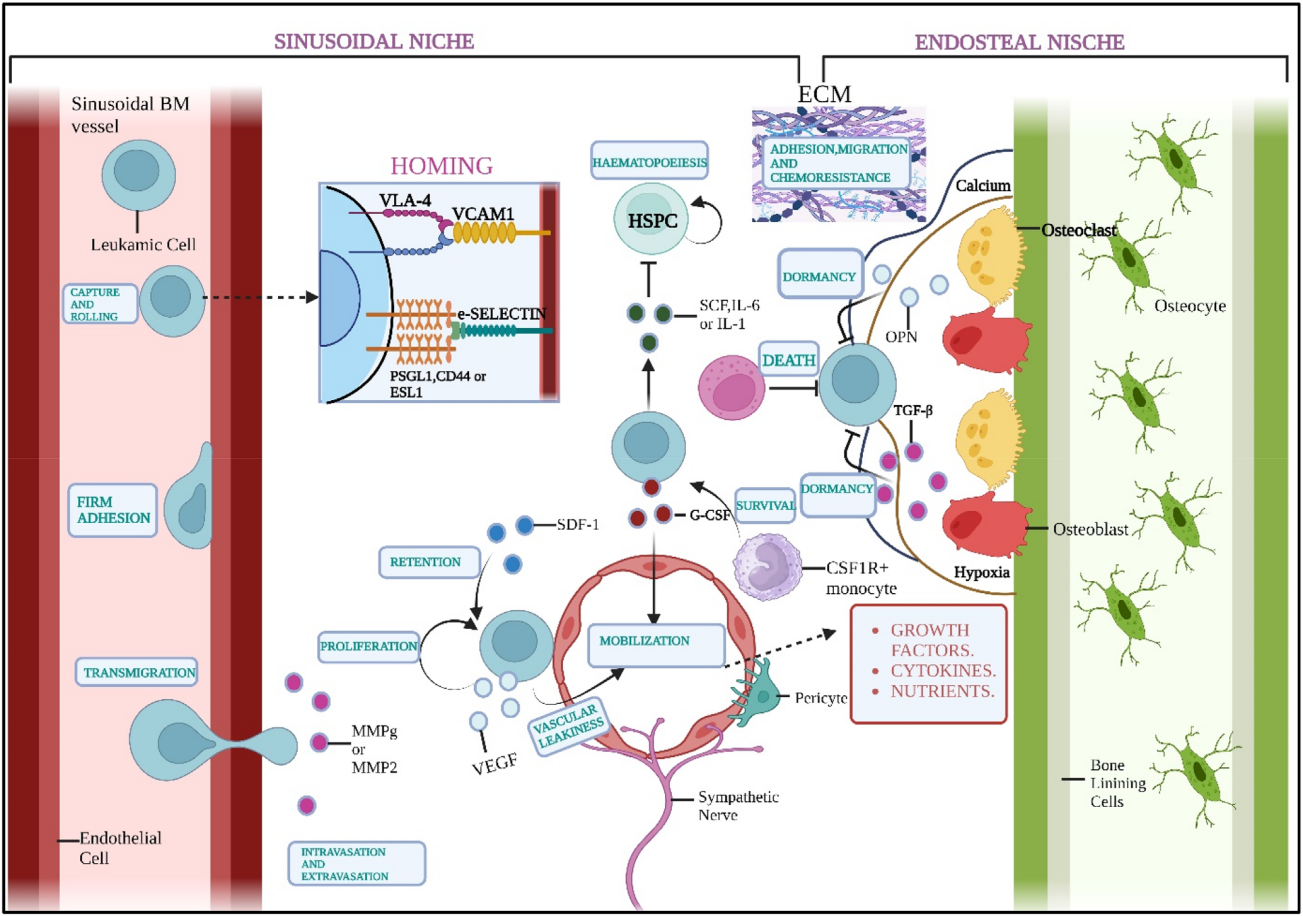
Metastasis in the Bone Marrow (BM). Chemokine stromal cell‐derived factor 1 (SDF1) regulates the homing of the cells. Integrin, VLA‐4 and e‐Selectin assist it. In the sinusoidal niche, leukaemia cells develop and thrive under the influence of stromal cells, pericytes and colony‐stimulating factor 1 receptor‐positive (CSF1R+) monocytes. Leukaemia cells destroy haematopoietic stem cells and progenitor cells (HSPCs) by Stem cell factor (SCF). This hampers normal haematopoiesis and leads to the formation of a malignant niche. Leukaemia cells might also be the endosteal niche. Here, the oxygen‐limiting environment, osteopontin (OPN) and Transforming Growth Factor –β (TGF‐ β) enable the leukaemia cells to undergo a dormant state. This state resists chemotherapy. Granulocyte Colony Stimulating Factor(G‐CSF) aids in metastasis by animating the cancer cells. Enzymes like elastase and matrix‐metallo proteinase 2 and 9 (MMP2 and 9) may initiate extravasation. Natural Killer (NK) Cells might fight against the blood cancer cells in BM (Created in Biorender.com).

**FIGURE. 4:**
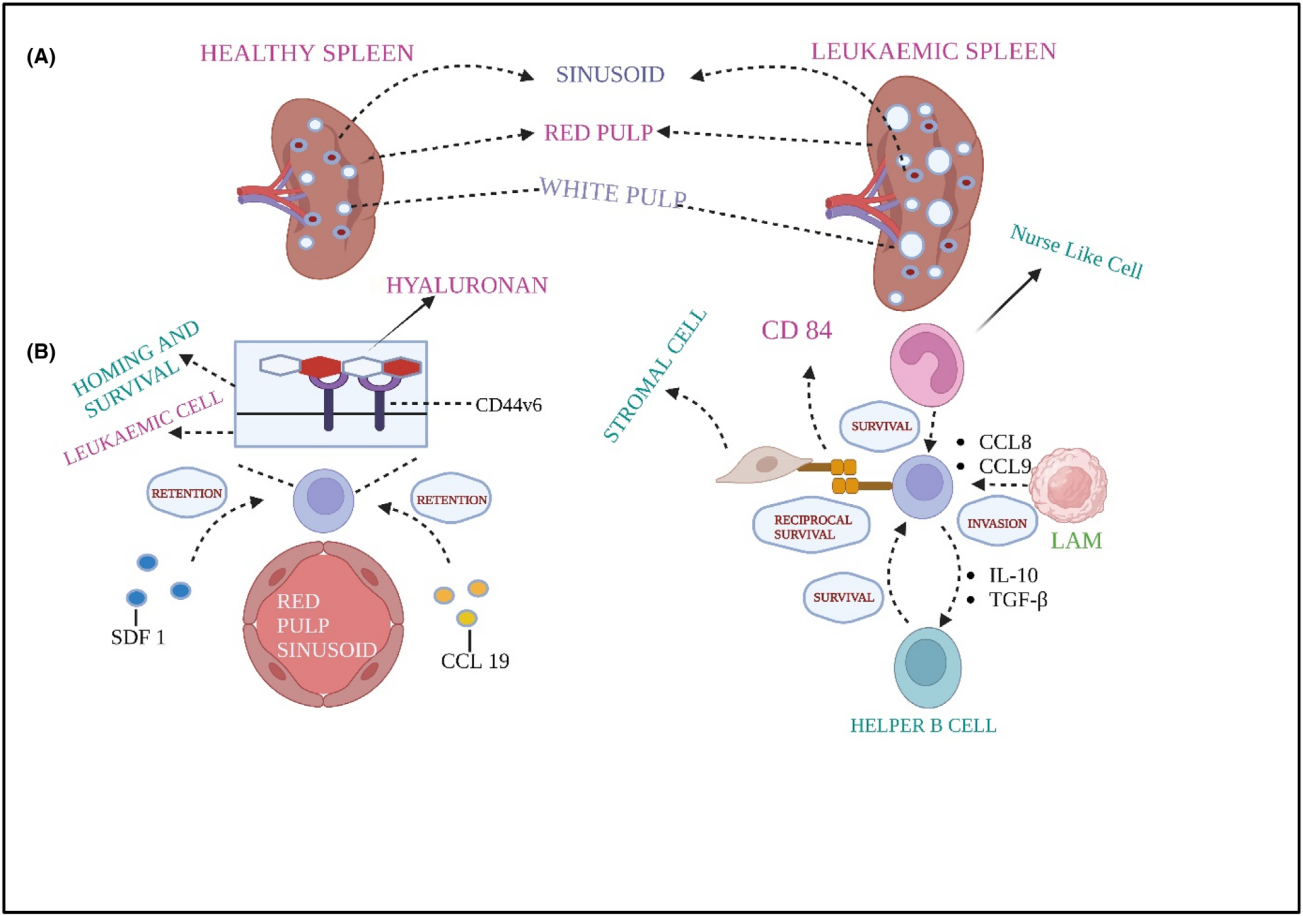
Metastasis in the Spleen. Usually, metastasis occurs here as leukaemia becomes chronic. Growth of the white pulp is responsible for a hypertrophic phenotype exhibited by several individuals suffering from blood cancer. Entry of the Leukaemia cells through the sinusoidal vessels is regulated by CCL1‐CCR7 and Stromal Cell‐derived Factor‐1(SCF‐1)‐CXCR4. Ly6C+ leukaemia‐associated macrophages (LAMs) might aid leukaemia cells moving to the spleen by CCL8 and CCL9. Hyaluronan, monocyte‐derived nurse‐like cells, helper B cells and many other stromal factors aid the cancer cells in multiplying and thriving. (TGF‐ β: Transforming Growth Factor –β) (Created in Biorender.com).

Metastasis is a defining feature of cancer and the primary reason for deaths related to cancer. In solid tumours, metastasis refers to developing tumours in an area of the body away from the actual cancer site.^80^ The process of metastasis involves cancer cells breaking away from their primary site, entering the bloodstream, surviving blood vessel pressure, adapting to the new environment in a distant location, and evading attacks from the immune system to create secondary tumours.^81^ The location of origin in the BM, LN, or spleen is frequently uncertain in liquid tumours like leukaemias that initially appear with widespread disease (Refer to Figures 3 and 4). Leukaemia's status as a metastatic illness has been debated due to this uncertainty and the realization that leukaemic cells ‘inherit’ motility rather than developing it gradually. Despite this debate, metastasizing solid tumour cells and cells from disseminated leukaemia often have much in common. Leukaemia metastasis is characterized by an incessant and conspicuously efficacious cycle of tissue homing, colonization and mobilization back into circulation. This makes leukaemia a challenging and life‐threatening condition and, in the case of acute leukaemias, an extreme example of aggressive metastasis.^82^ Research has shown that the specific characteristics of the initial cancer cell that enters the bloodstream play an essential part in determining the different properties of metastasis, like growth and response to treatment.^83^ In vivo and in vitro studies have demonstrated that cancer cells exhibit individual migratory behaviour.^84^ In humans, metastasis is believed to require the collective activity of a bunch of tumour cells that move together, which involves epithelial‐mesenchymal transition (EMT).^85^ Through EMT, transdifferentiated epithelial cells acquire the authority to seize, withstand stress and metastasize.^86^ Since epithelial cells are fixed and firmly connected and the ECM surrounding them,^87^ EMT controls the temporary biochemical changes that permit a particular epithelial cell to gain a mesenchymal phenotype, granting epithelial‐mesenchymal plasticity essential for cancer development and dissemination.^88^ Nevertheless, all the cells in the tumour site are not responsible for metastatic growth. Asparagine synthetase, a metabolic enzyme, has been linked to the formation of metastatic growth in a mouse model, as research on factors affecting metastatic potential has shown.^89^ Lowering asparagine levels with asparaginase therapy or dietary restriction slowed metastatic spread.^89^ The presence of asparagine encouraged EMTs, promoting metastasis. Intermediary stages with invasive, metastatic, and differentiation traits alternate spontaneously during EMT in primary tumour cells.^90^ Tumour cells exhibiting a combination of epithelial and mesenchymal phenotypes enhance circulation, colonization at the secondary site and movement to other sites.^90^ Post‐translational and epigenetic modulators also regulate the EMT process.^91^ While EMT is a well‐studied process in solid tumours, its role in leukaemia is less direct but still relevant. EMT refers to the biological process where epithelial cells acquire mesenchymal traits, enhancing their migratory and invasive abilities. This helps cytoskeletal reorganization.^92^

Although leukaemic cells are not epithelial in leukaemia, they can exhibit EMT‐like changes that enhance their invasive potential. For instance:

1. *Adhesion Molecule Modulation*: Alterations in the expression of adhesion molecules such as integrins and selectins enable leukaemic cells to detach from the BM niche and migrate to other tissues.
2. *Enhanced Mobility*: Leukaemic cells can reorganize their cytoskeleton, aiding their ability to traverse endothelial barriers and infiltrate other organs.
3. *Resistance to Apoptosis*: EMT‐like changes can also confer resistance to apoptosis, allowing leukaemic cells to survive in various microenvironments.

Thus, while leukaemia does not metastasize in the same manner as solid tumours, the spread of leukaemic cells to various organs poses significant clinical challenges. Understanding the mechanisms of EMT‐like changes in leukaemic cells and their organ‐specific homing behaviours is crucial for developing targeted therapies. Effective management of leukaemia requires addressing both the primary disease and the complications arising from organ‐specific infiltration, aiming for comprehensive treatment strategies that improve patient outcomes.^82^

More scientific studies are required to fully and precisely explain the function of EMT in cancer proliferation and metastasis.^80^

## 8 DRUG AND THERAPEUTIC RESISTANCE

Drug and therapeutic resistance are problematic when diseases get acquainted with pharmaceutical treatments. Earlier, numerous cancer types could be treated with chemotherapy. However, as time progresses, they become tolerant and acquire a lack of sensitivity to the current treatment methods through DNA mutations and metabolic changes, which assist the reticence of drugs. Drug resistance is a significant barrier to treating acute leukaemia. It may be brought on by many mechanisms, like:

1. The expression of drug efflux pumps that reduce the intracellular concentration of chemotherapeutic drugs. Leukaemic cells often express high levels of ATP‐binding cassette (ABC) transporters, such as P‐glycoprotein (P‐gp), which pump chemotherapeutic drugs out of the cells, reducing intracellular drug concentration and efficacy.^93^

2. The switching of DNA repair pathways that mend the harm inflicted by chemotherapeutic drugs.^94^ Epigenetic alterations, mainly DNA methylation and histone modifications, can deactivate genes involved in drug sensitivity. These changes are reversible, indicating possible therapeutic targets to combat resistance. ^94^

3. Mutation and amplification of the genes that code for pharmaceutical targets and signalling molecules. Leukaemia cells frequently develop mutations that allow them to survive chemotherapy. For example, mutations in the BCR‐ABL gene in CML can cause resistance to tyrosine kinase inhibitors (TKIs) like imatinib by altering the drug's binding site, reducing its effectiveness. Some genes may be amplified, leading to increased production of proteins that cause resistance. For instance, amplifying the MDR1 gene, which encodes P‐glycoprotein, can expel chemotherapeutic drugs from cells, lowering their intracellular concentrations and effectiveness.^93^, ^95^, ^96^

4. Drug inactivation. Changes in the structure or expression of drug targets can make treatments ineffective. In ALL, alterations in the CD22 gene can result in resistance to immunotoxins targeting this protein.^97^

5. Programmed Cell death (Apoptosis) inhibition. Leukaemic cells can evade apoptosis through overexpression of anti‐apoptotic proteins like Bcl‐2 or downregulation of pro‐apoptotic factors such as Bax, thereby surviving despite drug treatment.^98^

6. The EMT against current treatment methods.

7. Cell heterogeneity and certain epigenetic modifications in malignant tumours.^99^ Changes like DNA methylation and histone acetylation can modify gene expression, leading to drug resistance. Epigenetic alterations that silence tumour suppressor genes or activate oncogenes can enhance the survival and proliferation of leukaemic cells.^100^

The Bone Marrow Stromal Cells (BMSCs), used to deliver therapeutic agents to the tumour sites, can aid in drug resistance. The exosomes secrete granules that modulate drug resistance by communicating with drug molecules. Exosomes facilitate cell‐to‐cell contact in the TME and can make tumour cells resistant to drugs by transferring specific mRNAs, ncRNAs or proteins.^101^ Exosomal granules change the cancer cells' transcriptome, affecting immune activity. The involvement of the exosomes in drug resistance might differ depending upon its contents, including nucleic acids (DNA or RNA), proteins, lipids, metabolites, etc. These exosomes are primarily acquired from the Cancer‐associated fibroblast (CAF), and drug‐sensitive cells can take up those drug‐resistant tumour cells. As a result, the exosomal cargos (containing DNA, RNA and proteins) play a crucial role in transferring drug resistance to the cells that are sensitive to drugs.^102^ Both naive and 5 T33 BMSC‐derived exosomes accelerated the proliferation of MM cells and brought on bortezomib drug resistance. Experiments have revealed that exosomes obtained from BMSCs can affect the initiation of many survival‐related pathways, including p38, p53, c‐Jun N‐terminal kinase and Akt, in a similar manner.^103^ A crucial component of AML therapeutic resistance is the interaction between BMSCs and AML, which secretes growth factors, cytokines and EVs. Exosomes, secreted AML cells, act as vital messengers between BMSCs and AML. According to a previous study, they have been shown to protect AML cells from chemotherapy‐influenced apoptosis. Drug resistance in leukaemia can be overcome using different methods, namely‐ Using inhibitors to prevent the action of DNA repair enzymes or drug efflux pumps,^94^ Combining different drugs that target different pathways or mechanisms,^95^, ^96^ and keeping track of the molecular reaction and modifying the course of treatment as necessary.^96^, ^99^

## 9 SOURCE OF BIOMARKER

One of the advantages of exosomes as biological carriers is that the miRNAs (microRNAs) derived from exosomes are resistant to degradation by extracellular ribonucleases. This property makes exosomes a repeatable and reliable biomarker (Refer to Table 1). According to some studies, exosomes, particularly their miRNAs, can persist steadily and significantly in various bodily fluids, including blood and urine.^104^ Experimental evidence shows that certain exosomal proteins and miRNAs and their types and expression levels are directly linked to leukaemia. It is vital to gather bodily fluids and assess the pathology under non‐invasive conditions to check the proliferation of the disease because exosomes from diseased cells have comparable miRNA expression profiles to the parental cells.^105^ Thus, exosomes are suitable for use as non‐invasive biomarkers. Using the actin structure in ALL‐produced exosomes as a reference, scientists devised a technology for tracing ALL‐derived exosomes in patients' blood. This gave a new way to diagnose, therapy and anaesthesia in children affected by ALL. Exosomes have been thoroughly studied as potential CLL indicators. The miRNA155 and the exosomes CD19, CD37 and CD52 are valuable markers for CLL clinical staging and drug recommendations. In addition to transferring the associated miRNAs to healthy mesenchymal stromal cells, exosomes comprising CLL transcripts can promote cancer growth. Studies have revealed that exosomes obtained from CLL can modulate the tumour microenvironment by controlling Akt signalling and promoting the lofty expression of CLL promoters, including VEGF (vascular endothelial growth factor). The increase in the quantity of expression of VEGF can be implemented as a biomarker.^106^ The surface of exosomes collected from cancer cell culture media and the plasma of CLL patients were examined in a study utilizing a DotScan antibody microarray to track the development of cancer and dynamically track the deterioration of the condition of patients.^107^ Exosome variations in protein and/or TGF‐ β 1 level may also indicate how chemotherapy has affected AML patients. Plasma exosomes may indicate that patients in complete remission still have residual lesions. After treatment, low exosome levels suggest long‐term survival without illness. Therefore, exosomes derived from plasma can be utilized as a potential biomarker to track leukaemia relapse in AML patients as a measure of the effectiveness of chemotherapy.^108^ According to a study on haematological malignancies, AML patients had much higher levels of TGF‐ β exosomal expression than healthy individuals. TGF‐β levels dropped significantly following treatment. TGF‐ β levels are very low during long‐term complete remission, which raises the possibility that exosomes from AML could be employed as a therapeutic sign.^109^ Different research showed that the levels of EVs in the circulating blood of individuals with AML were significantly elevated at three different phases of the disease (initial diagnosis, neutropenia and remission) when compared to the individuals with total remission and healthy individuals. These findings suggest that EV levels may serve as an indicator for the detection of minimal residual disease, aiding in diagnosing and monitoring AML.^110^ Experiments conducted on AML mouse models have exhibited that exosomes derived from the disease can lead to the downregulation of SCF and CXCL12 in haematopoietic stem cells within the stromal environment. These findings show that exosomes act directly and indirectly in suppressing leftover haematopoietic function before leukaemic cells' widespread infiltration of the BM.^111^ Research to understand haematological malignancies found that miR‐155 levels in microparticles may serve as a pre‐post marker for CLL patients and a risk marker for developing CLL from monoclonal B lymphocytosis.^112^ The presence of CD44 in serum EVs from individuals suffering from multiple myeloma (MM) has also been found to be a possible measure for evaluating overall survival, that is, prognosis.^113^ Exosomal miRNAs may be utilized as a therapeutic indicator because it has been demonstrated that some exosomal miRNAs are differentially expressed in individuals (both high and low‐risk) suffering from MM.^109^

**TABLE 1:** Diagnostics and therapeutic exosome biomarkers in leukaemia.

Biomarker	Source	Exosome molecule	Clinical signature	References
Diagnostic	CLL plasma	miR‐29a‐c, miR‐150, miR‐155	Compared to healthy donors, CLL patients have shown considerable over‐expression in their plasma exosomes.	^114^
	CLL cells	miR‐202‐3p	Over‐expression has been found in the plasma exosomes of patients suffering from AML as compared to healthy donors.	^115^
	BMMSCs	miR‐23b‐5p, miR‐339‐3p, miR‐425‐5p	Considerable downregulation in MSCs‐Exo of AML patients compared to healthy donors	^116^
	BMMSCs	miR‐101‐3p	Considerable overexpression in the MSCs‐Exo of patients suffering from AML as compared to the donors who are healthy	^116^
	Chronic myeloid leukaemia (K‐562), colorectal carcinoma (HCT116) and murine melanoma (B16‐F10) cell lines	Double‐stranded DNA	It can act as an alternative to tumour tissues.	^117^
	AML Myeloid cell	NPM, FLT3, MMP9, IGF‐I, CXCR4	These particular biomarkers are present in exosomes derived from AML cells	^118^
Prognostic	AML plasma	miR‐532	Hefty amounts of exosomal miR‐532 are related to overall survival	^119^
	AML serum	miR‐125b	Hefty amounts of exosomal miR‐125b are related to a higher mortality rate in moderate‐risk AML.	^120^
	CML plasma	miR‐320	Considerably overexpressed in plasma exosomes of CML‐BC patients compared to patients suffering from CML‐CP	^121^
	CML plasma	miR‐215	Downregulated exosomal miR‐215 shows successful discontinuation of imatinib	^122^
	CML cells	circ_0058493	A large level of exosomal circ_0058493 is related to IM resistance	^123^
	CLL plasma	circRNA mc‐COX2	Great levels of exosomalcircRNA mc‐COX2 are related to CLL proliferation and forecasting	^124^
	AML Myeloblast	miR‐4532	Exosomal miR‐4532 inhibits general blood cell production	^125^
	Tumour cells of many types of cancer, like CLL	S100 protein	Detection of exosomal S100 protein is related to the advanced‐stage disease	106, 126

## 10 THERAPEUTIC APPROACH

**FIGURE 5:**
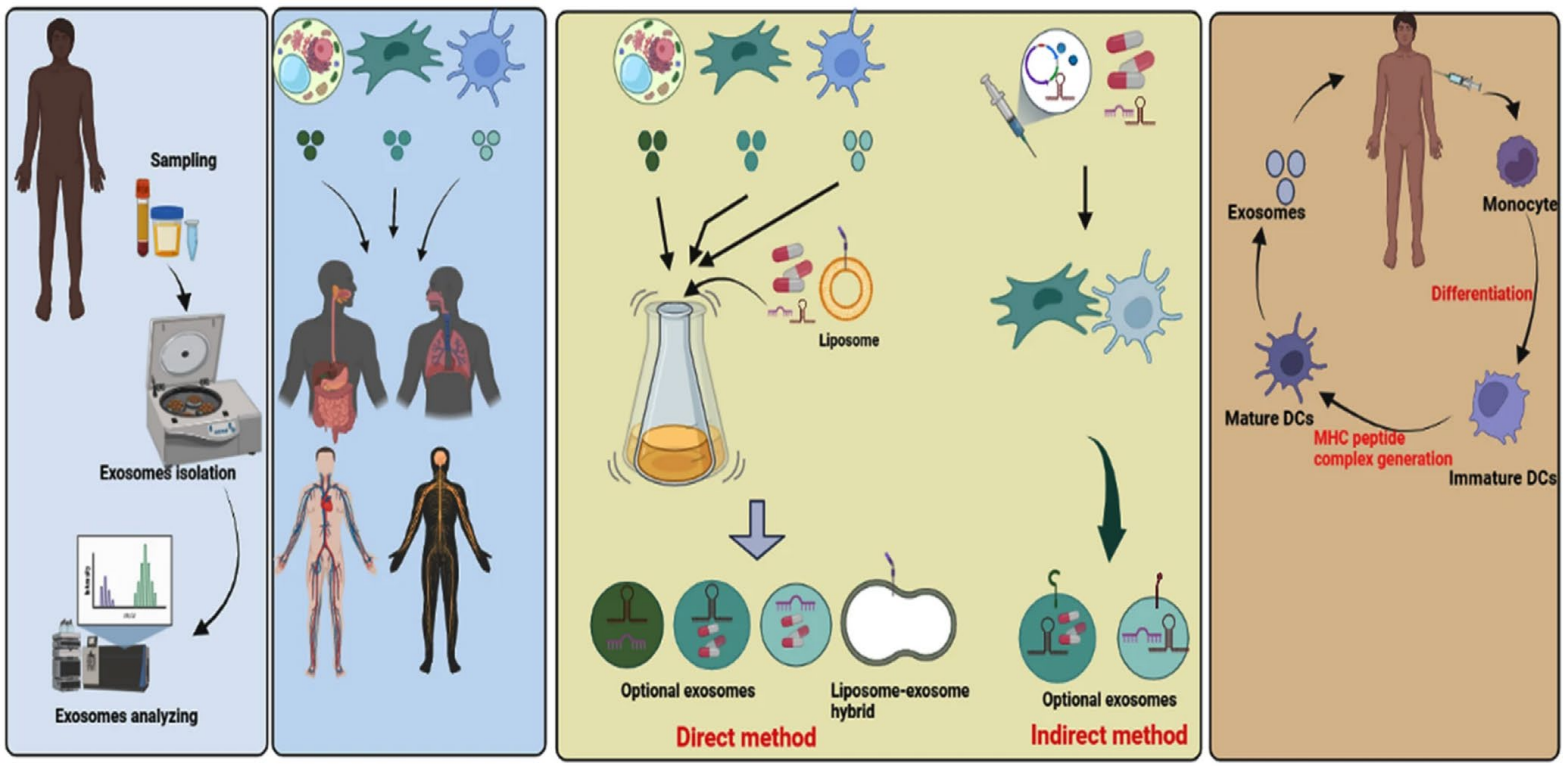
The therapeutic utilization of exosomes encompasses various applications in clinical trials, including their role as biomarkers, cell‐free therapy (exosome therapy), carriers for drug delivery and cancer vaccines. Exosomes derived from plant cells, mesenchymal cells, T cells and dendritic cells are employed to treat diverse ailments. Furthermore, exosomes derived from these sources exhibit promise as vehicles for drug delivery systems. In the direct approach, therapeutic agents are loaded into exosomes. In contrast, the indirect methods involve genetic engineering or co‐culturing of specific cells with therapeutic agents to generate artificial exosomes (Adapted with permission from ref^134^ Copyright © 2022, The Author(s)).

Exosomes are small vesicles released by cells showing great promise as a therapeutic vehicle for leukaemia‐related malignancy. Experiments have exhibited that exosomes taken from MSCs can enhance the clinical condition of patients suffering from graft‐versus‐host disease (GVHD) by modulating the activity of the mononuclear cells present in the circulating blood of the patient and their immune response to inflammatory cytokines. This effect is observed soon after therapy.^127^ Exosomes acquired from various sources have also effectively delivered drugs, miRNAs and antigens to target cells in haematological malignancies and cross the BBB to treat CNS‐associated leukaemias.^128^ However, exosomes have also been associated with developing venous thrombolytic events in haematological malignancies.^129^ Exosomes can bind with tissue factors, which can instigate the coagulation cascade and eventually result in blood clot formation. Accumulation of pro‐coagulant exosomes in the cancer patient's plasma has proved to be an inferior factor for prognosis. It can be misleading in the clinical diagnosis of patients. Ongoing clinical trials focus on eliminating or decreasing the levels of pro‐coagulant exosomes as a therapeutic approach. This is being done because exosomes can trigger blood clot formation.^130^ To maximize the therapeutic potential of exosomes while minimizing their potential adverse effects, researchers are developing methods to discriminatingly silence exosome‐delivered messages that encourage cancer development and inhibit exosome‐mediated immune subsidence. Small‐molecule inhibitors like ALIX can attenuate exosome biogenesis, while intracellular Ca^2+^ levels were found to modulate exosome deployment in K562 CML cells.^131^ Na^+^/Ca^2+^ exchange inhibitor dimethyl amiloride has been shown to reduce exosome secretion and suppress tumour development in mouse and human cancer models.^132^ (Refer to Figure 5). In summary, while exosomes hold great promise as a therapeutic tool for leukaemia‐related malignancy, efforts are ongoing to address their potential adverse effects, such as developing venous thrombolytic events and promoting immune subsidence in recipient cells.^133^ Potentially, the future of exosome research is bright.

## 11 CLINICAL TRIALS

Clinical trials play a crucial role in advancing our understanding of cancer, and in recent years, researchers have turned their attention to the potential of exosomes in the context of bone cancer. Exosomes, small vesicles released by cells, contain RNA and proteins that may serve as valuable biomarkers and therapeutic targets. Clinical trials for exosomes in the diagnosis and therapeutics of Leukaemia are mentioned in Table 2.

**TABLE 2:** Summary of exosomes in leukaemia theragnostic (source: cliNical trials.gov.in).

Exosome source	Clinical ID	Clinical Significance
PB and bone marrow samples	NCT04460963	Role of adrenomedullin in leukaemic
Blood	NCT03283228	Cd11b and Cd56 as Prognostic Markers
Blood	NCT03743909	Incidence of expression of aberrant CD markers in acute leukaemia
Blood	NCT01540578	Biomarkers as a diagnostic tool in samples from younger patients
Bone marrow	NCT01298414	Biomarkers in bone marrow samples from young patients
Blood and bone marrow	NCT01005368	Study of biomarkers in blood and bone marrow samples from patients with previously untreated chronic lymphocytic leukaemia
Blood	NCT03249636	New markers for minimal residual disease
Tissue	NCT01229124	RNA biomarkers in tissue samples from infants
Bone marrow	NCT02527447	Biomarkers for personalized early assessment of response during salvage chemotherapy

## 12 FUTURE PROSPECTIVE

In leukaemia, exosomes have exhibited potential as therapeutic agents and diagnostic tools. Exosomes can be engineered as therapeutic agents to carry specific payloads, such as cytotoxic drugs or targeted therapies, allowing for focused delivery to leukaemic cells. This targeted delivery system can enhance the effectiveness of treatments while minimizing side effects.^135^ Additionally, exosomes can transport gene‐editing tools like CRISPR‐Cas9, providing a novel approach to directly modify leukaemia‐associated genes and potentially eradicate the disease at its genetic core. On the diagnostic front, exosomes hold great potential as biomarkers for leukaemia.^136^ They are released by blood cancer cells and can be acquired from several bodily fluids. The cargo within exosomes, including specific proteins or genetic material, can supply a profound understanding of the state of the disease, prognosis and reaction to treatment.^108^, ^109^, ^137^ Analysing exosomes allow non‐invasive and real‐time disease progression monitoring, enabling early relapse detection and guiding personalized treatment strategies.^109^ Furthermore, exosomes can aid in liquid biopsies, which are less invasive than tissue biopsies.^68^ They can provide a comprehensive profile of leukaemia, capturing its heterogeneity and evolutionary patterns over time. This information can assist in making treatment decisions, allowing healthcare professionals to tailor therapies based on individual patient characteristics.

## 13 CONCLUSION

The potential impact of exosomes in leukaemia therapy and diagnostics is poised to revolutionize cancer treatment. The ability of exosomes to transport targeted therapies, facilitate genetic modifications, and act as biomarkers holds tremendous promise for improving patient outcomes. As research in exosome biology progresses, we can envision a future where exosome‐based therapies and diagnostics become essential components of leukaemia management, providing more effective and personalized approaches to combat this challenging disease.

## AUTHOR CONTRIBUTIONS

**Subhrojyoti Ghosh:** Conceptualization, Data Curation, Formal Analysis, Writing – original draft. **Anuvab Dey:** Data Curation, Formal Analysis, Writing – original draft. **Aneshwa Chakrabarti:** Data Curation, Formal Analysis, Writing – original draft. **Tiyasa Bhuniya:** Data Curation, Formal Analysis, Writing – original draft. **Neelparna Indu:** Data Curation, Formal Analysis, Writing – original draft. **Anirban Hait:** Data Curation, Formal Analysis, Writing – original draft. **Ankita Chowdhury:** Data Curation, Formal Analysis, Writing – original draft. **Aritra Paul:** Data Curation, Formal Analysis, Writing – original draft. **Atharva A. Mahajan:** Data Curation, Formal Analysis, Writing – original draft. **Marios Papadakis:** Supervision, Funding Acquisition, Writing – review & editing. **Athanasios Alexiou:** Conceptualization, Supervision, Writing – review & editing. **Saurabh Kumar Jha:** Writing–review & editing, Visualization, Conceptualization.

## FUNDING INFORMATION

Open Access funding enabled and organized by Projekt DEAL. This work was supported by the University of Witten‐Herdecke Germany.

## CONFLICT OF INTEREST STATEMENT

The authors declare that they have no competing interests.

## CONSENT

Not applicable.

## Data Availability

All data generated or analysed during this study are included in this published article.
